# Typhoid Fever

**Published:** 1860-04

**Authors:** 


Typhoid Fever—Nathan Smith, &c.—Dr. Bowling, of the *Nashville Journal*, for March, is anything but "genial." The good humored comments of "A," upon his proposed treatment of typhoid fever, have given rise to an exacerbation of unpleasant emotions alarming in the last degree. For the use of the term "inventor," which he re-produces in capitals, and garnishes with an exclamation point—

> "Which stands in state, with vacant wonder fraught,
> The pompous tombstone of a pauper thought."

We confess to have relied upon Ainsworth, Webster, and Johnson. It appears that our amiable critic relies for his definition upon the Patent Office—does he propose to take his medicines from or to the same quarter? We yield to no man in admiration for the talents and acquirements of Nathan Smith. He needs no eulogium from "A," neither from the senior editor of the *Nashville Journal*, although that senior editor claims peculiar powers of "writing up," whoever will bow to his behests. *Does Dr. Bowling understand?*

As a matter of history, every New Englander will recollect that from the great use of that particular article (buttermilk) by Dr. Smith, as an article of diet and diluent, in typhoid fever, his practice was familiarly termed "the buttermilk practice," by both opponents and friends.

We have yet to learn that it is necessary to endorse every particular of doctrine and practice adopted by any eminent medical gentleman, in order to show that we worthily recognize his merit. In all humility we confess that we do not even do this for the senior editor of the *Nashville Journal*.

